# A Case of Perforation of Bladder

**Published:** 1874-08

**Authors:** L. J. Willien

**Affiliations:** Terre Haute, Indiana


ARTICLE VI. *A Case of Perforation of the Bladder, followed by Recovery.* By L. J. WILLIEN, M.D., Terre Haute, Indiana.

Daniel S——, æt. twenty years; sanguino-lymphatic temperament, strong constitution, light complexion, medium size; miner by profession; born in Scotland; working in the mines at Brazil, Indiana, some three years; enters Providence Hospital on the 27th day of November, 1872. Being summoned to his room, we obtained the following history of the case:

Six weeks previous to his arrival at the hospital, while working in a mine, one of the arches in the gangway gave way; a large block of slate felled him to the ground, striking the lower portion of the spinal column, pelvis, and lower extremities; he was then removed to his boarding house, with a complete paraplegia; beginning about the third lumbar vertebræ, both motion and sensitiveness were lost, as also the bladder and rectum. Although no evident signs of fracture of the pelvis were discovered, yet internal injuries were to be feared.

The attending physician, perhaps not forseeing the dangerous consequences, introduced a silver catheter into the bladder, and left it there without removing it for several days, and the patient apparently regained motion and sensibility of the parts injured. Suddenly he was taken with intense burning pains in the lower bowels, repeated chills, with high fever, followed by an abundant perspiration, with tumefaction extending from the pubis up to the right crest of the ilium, and descending along the groin to the lower third of the thigh.

After undergoing a general treatment, both internally and locally, the abscess broke about two and a half inches below the crest of the ilium, discharging a vast quantity of thin pus and urine. Two weeks from that time Daniel was sent to the hospital by his friends in such a weak and exhausted condition that they feared he might not reach the hospital alive. On his arrival he presented the following symptoms: Great emaciation of the body; skin dry and hot; eyes sunken in their orbits; tongue red and dry; breathing short and quick, with numerous mucous and sibilant rales disseminated through the posterior parts of the chest; great thirst; no appetite; pulse weak and depressible, one hundred per minute; bowels constipated; passes no urine through the natural channels; part motion and sensibility in the left leg, with total absence of same on the right side. All inflammation, excepting of the thigh, had subsided, leaving the fistulous opening already mentioned, out of which freely flowed a heavy stream of urine when direct pressure was made upon the bladder. The thigh was very painful, surface of the skin red, with evident fluctuation. As far as probing would permit us through the fistula, we had before us a plain case of perforation of the bladder of the middle and anterior surface, causing numerous urinary abscesses of the abdominal muscles and thigh. Two indications presented themselves as to the treatment of the case: 1st. A good nutritious diet, such as beef essence, rare steak, eggs, etc., wine, egg-nog, etc. Medicines: Tincture of cinchona comp., one teaspoonful three times a day. Externally: Carbolic acid, one-half drachm, rain water, oz. iv. Mix, and apply to fistula. At the same time we introduced a small catheter bougie as far as possible with safety, and pushed with a syringe some of the solution through the fistula, besides introducing a soft rubber bougie into the bladder, to favor a free evacuation of urine. Applied warm poultices, well sprinkled with laudanum.

Nov. 28th. Same condition; same treatment; bowels constipated. Ordered five grains pulv. rhubarb every three hours until effect. Made three free incisions into the thigh to evacuate accumulated pus and urine; then made free injections through the diffused abscess with tepid carbolized water, ordering the whole covered with a poultice.

Nov. 30th. Patient somewhat better; pulse eighty per minute, soft and regular; cough less severe. R. Syrup phosph., iron, quin. and strychn., tr. cinchon. comp., *aa* dr. j. Repeat three times daily, and continue same as before.

Dec. 2nd. Same state and same treatment. Same on Dec. 3rd, 4th, 5th, 6th and 7th, with slow improvement.

Dec. 10th. Urinates without catheter; the abscess of the thigh heals rapidly; very little urine escapes through the fistula; his appetite is good; continue same treatment.

Dec. 18th. Urinary fistula nearly closed; no more urine escapes; thigh well, and patient asks permission to leave the bed, having no remaining symptoms of paraplegia. We continued a tonic treatment and diet during two weeks more, without intermission, after which time the patient had entirely recovered.

